# Electrochemical Performance of Biopolymer-Based Hydrogel Electrolyte for Supercapacitors with Eco-Friendly Binders

**DOI:** 10.3390/polym14204445

**Published:** 2022-10-20

**Authors:** Giovanni Landi, Luca La Notte, Alessandro Lorenzo Palma, Giovanni Puglisi

**Affiliations:** ENEA, Casaccia Research Center, Via Anguillarese 301, 00123 Rome, Italy

**Keywords:** water processable, sustainable binder, gelatin, hydrogel electrolyte, carbon-based supercapacitor, pseudocapacitive material, charge storage mechanisms, cycle stability

## Abstract

An environmentally friendly hydrogel based on gelatin has been investigated as a gel polymer electrolyte in a symmetric carbon-based supercapacitor. To guarantee the complete sustainability of the devices, biomaterials from renewable resources (such as chitosan, casein and carboxymethyl cellulose) and activated carbon (from coconut shells) have been used as a binder and filler within the electrode, respectively. The electrochemical properties of the devices have been compared by using cyclic voltammetry, galvanostatic charge/discharge curves and impedance spectroscopy. Compared to the liquid electrolyte, the hydrogel supercapacitors show similar energy performance with an enhancement of stability up to 12,000 cycles (e.g., chitosan as a binder). The most performant device can deliver ca. 5.2 Wh/kg of energy at a high power density of 1256 W/kg. A correlation between the electrochemical performances and charge storage mechanisms (involving faradaic and non-faradaic processes) at the interface electrode/hydrogel has been discussed.

## 1. Introduction

The technological advances in consumer electronics and the rapid diffusion of related products in our daily lives increase energy demands [1]. Electrochemical energy storage, including batteries and supercapacitors, is the most practical and flexible strategy for portable power devices for a plethora of applications and the industry is focused on developing more efficient and cost-effective products [2,3,4]. The state-of-the-art Lithium-Ion Batteries deliver a high specific energy density of 250–270 Wh kg^−1^ and a relatively low specific power density (<350 W kg^−1^) [5]. In contrast, commercial supercapacitors show a high power density of up to 10 kW kg^−1^, but they have a low specific energy density (<10 Wh kg^−1^) [6]. However, these energy sources contribute to the increment of electronic waste that poses environmental concerns due to heavy metals and brominated flame retardants in plastics [7] and the challenging recovery of valuable metals [8]. Thus, the design and development of new electrochemical storage systems must consider abundant and safe materials and sustainable production processes. Electrochemical capacitors or supercapacitors (SCs) have suitable features in terms of performance (high power density and long cycle life), versatility (shape, size and lightness) and environmental friendliness to deliver power for modern and sustainable electronics [9,10,11]. In particular, carbon-based SCs offer the opportunity to be fabricated from natural-derived materials or industrial by-products. Great efforts by the research community have been devoted to the components of supercapacitor electrodes in order (i) to produce activated porous carbon from plant biomass [12,13] and (ii) to replace the fluorinated materials (poly(vinylidene difluoride (PVDF) and polytetrafluoroethylene (PTFE)) generally used as binders with biopolymers processable in water [14,15].

The choice of electrolyte is the key to achieve high and stable supercapacitor performances. Based on different solvents, the electrolyte can be aqueous, organic or ionic liquid. Because of their high voltage window (2.6 to 2.9 V), devices based on organic electrolytes are currently in the lead at commercial market [16], but they suffer from being flammable, volatile and toxic. Ionic liquids show even wide voltage windows up to 4.0 V but exhibit unsatisfactory conductivity and high viscosity, which results in poor rate performances, especially at low temperatures [17]. Furthermore, organic and ionic liquids are moisture-sensitive electrolytes, thus requiring complex and ultradry manufacturing procedures for the fabrication of SCs. The application of aqueous electrolytes represents the more sustainable and low-cost strategy [18], but as any liquid electrolyte, it can easily leak and volatilize during packaging. Recently, gel electrolytes have attracted increasing attention towards the realization of solid-state SCs. Gel polymer electrolytes (GPEs) allow for multiple roles of the electrolyte, separator and binder in a SC to be fulfilled and are generally composed of a polymer as a matrix and an electrolyte salt to provide mobile ions [19]. 

Hydrogel based on synthetic polymer derived from petro-materials, such as poly(vinyl alcohol) (PVA) and poly(ethylene oxide) (PEO), has been widely investigated for energy storage applications due to their large intrinsic ionic conductivity value compared to the solid electrolyte [20]. Among these polymers, PVA has been the most examined because of its low cost, good electrochemical stability, good mechanical properties and non-toxic nature [21,22]. More recently, to reduce the dependence on fossil fuels and to improve the sustainability of the final devices, natural biopolymer-based gel obtained from renewable resources (e.g., gelatin, cellulose, guar gum, agarose, chitosan, DNA, etc.) is drawing much attention because of its large availability, low cost, biodegradability and lower environmental footprint [23,24,25,26,27].

In the present study, carbon-based supercapacitors were fabricated by incorporating sustainable binders (such as chitosan, carboxymethyl cellulose (CMC) and casein) and activated carbon (AC) within the electrode. Here, the AC material comes from the carbonisation process of the coconut shells. Moreover, the water-processable hydrogel electrolyte is based on a gelatin-glycerol blend doped with 2 M of NaCl. It is worth noting that gelatin is a biodegradable polymer obtained from the hydrolysis of the fibrous insoluble collagen present in bones and skin which is, currently, an abundant waste product of meat processing [28].

The fabricated devices have been fully characterized in terms of electrochemical performance and the advantages of using gel electrolytes with respect to liquid ones have been highlighted. A clear correlation between the cycle stability, charge storage mechanisms and dielectric properties at the interface electrode/hydrogel has been investigated in detail.

## 2. Materials and Methods

### 2.1. Materials Preparation

Supercapacitor test structures were fabricated onto polyethylene terephthalate (PET) foils (Melinex ST 504, DuPont Teijin Films, Chester, VA, USA, thickness 125 μm) covered with copper (Cu) tape (Kohree, City of Industry, CA, USA, thickness 40 μm). Henkel Electrodag PF407C graphite ink was deposited on the PET/Cu substrates by blade coating (Proceq ZAA 2300, Zehntner GmbH Testing Instruments, Sissach, Switzerland), and it was thermally annealed at 90 °C for 30 min, resulting in films 50 μm thick. The active material of the electrode was prepared by dissolving activated carbon (Kuraray YP 80F, Tokyo, Japan, with characteristic V_micro_ < 2 nm = 0.652 cm^3^/g and specific surface area (SSA) = 2093 m^2^ g^−1^) obtained from coconut shells and binder in ultrapure water (Milli-Q) according to the composition 95:5 wt.%. The investigated binders were CMC (Thermo Fisher, carboxymethyl cellulose sodium salt, Waltham, MA, USA), chitosan (Sigma-Aldrich, chitosan from shrimp shells, Saint Louis, MO, USA) and casein (TCI, casein sodium from milk). Among these biomaterials, chitosan needed an acidic solution to be dissolved; therefore, acetic acid was added to the formulation. The AC/binder mixture was stirred until a homogeneous carbonaceous slurry was obtained. Then, the slurry was deposited on the PET-Cu-Graphite stack by blade coating and dried at room temperature. At this stage, all the electrodes were weighed to obtain the AC mass loading which values were found to range from 6 and 6.8 mg/cm^2^. In total, 20 samples were prepared for each binder. The electrodes were sorted according to the $m_{a}$ mass and similar-mass electrodes were paired up for the supercapacitor devices. 

The electrolyte in the form of hydrogel was prepared by incorporating 2 g of gelatin (Sigma-Aldrich, gelatin from porcine skin) into 15 mL of an aqueous 2 M NaCl solution to achieve the highest gel electrolyte conductivity [29]. Successively, 1.5 mL of glycerol was added to the solution while stirring at 65 °C until complete dissolution of the gelatin powder. Glycerol acts as a plasticizer with the aim to reduce intermolecular forces in the gelatin network, thus increasing the mobility of polymeric chains and improving film flexibility [30]. To fabricate hydrogel at different molar concentrations of salt, an aqueous solution of 0 M and 2 M NaCl was used, respectively. The supercapacitor was completed by facing another electrode to form a sandwich structure. The electrode area A was 2.5 × 4 cm^2^.

### 2.2. Electrochemical Characterization

The electrochemical characterizations such as cyclovoltammetry (CV), galvanostatic charge–discharge (GCD) and electrochemical impedance spectroscopy (EIS) of the supercapacitors were measured on a commercial platform (Arkeo—Cicci Research) at room temperature. The devices were measured in a two-electrode geometry with an average area of about 10 cm^2^. The EIS measurements were performed in the frequency range between 100 mHz and 10 kHz with an ac-signal amplitude of 50 mV at open-circuit voltage. 

The gravimetric capacitance $C_{S}$ (F/g) of the symmetric SC has been computed by integrating the area under the CV curves according to the following equation [31]

(1) $$C_{S}=\frac{1}{m_{a}\cdot \nu \cdot \left(V_{b}-V_{a}\right)}\cdot \int_{V_{a}}^{V_{b}}i\left(V\right)dV$$

where $m_{a}$ is the mass of the electrode, *υ* is the scan rate, i(V) is the charging/discharging current and $V_{b}-V_{a}$ is the potential window. From the GCD profiles, the equivalent series resistance (ESR) can be estimated by

(2) $$ESR=\frac{IR_{drop}}{2\cdot I_{D}}$$

where $IR_{drop}$ is the voltage drop between the first two points of the discharge plot and *I_D_* is the discharge current. Energy E (Wh/kg) and the power P (W/kg) densities of the supercapacitors were computed by taking into account the equations

(3) $$E=\frac{1}{2}\cdot C_{S}\cdot {\left(\Delta V\right)}^{2}=\frac{1}{2}\cdot C_{S}\cdot \frac{{\left(V_{max}-V_{min}-IR_{drop}\right)}^{2}}{3.6}$$

and

(4) $$P=\frac{E}{t_{disc}}\cdot 3600$$

where $V_{max}$ is the maximum voltage applied to the device, $V_{min}$ is 0.1 V and $t_{disc}$ in seconds is the corresponding discharge period, respectively.

## 3. Results

**Figure 1:**
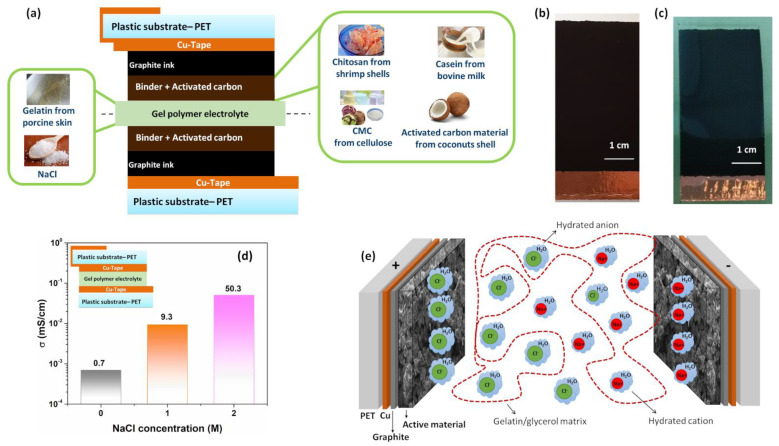
(**a**) Cross section of the symmetric carbon-based supercapacitors fabricated with sustainable materials; (**b**,**c**) photographs of the top view of the half structure of the device before and after the deposition of the transparent hydrogel, respectively; (**d**) ionic conductivity at 300 K as a function of the NaCl concentration within the hydrogel; (**e**) schematic diagram of a hydrogel polymer electrolyte (gelatin/NaCl/H_2_O) between two carbon electrodes. The cross section of the cell structure used to perform the conductivity measurements within the electrolyte is shown in the inset.

To evaluate the influence on the dielectric properties and cycle stability of environmentally friendly carbon-based supercapacitors with sustainable gel polymer electrolyte, test structures have been fabricated. Figure 1a shows the cross section of the device formed by a symmetric sandwich assembled following the sequence of the layers: PET/Cu-Tape/Graphite ink/Active material/Gel polymer electrolyte. The corresponding chemical structures for the sustainable binders and biopolymer electrolyte are reported in Figure S1a. The top views of the half structure, before and after the deposition of the transparent hydrogel, are displayed in Figure 1b,c, respectively. All the electrodes display relatively homogeneous and dense surfaces without significant holes or cracks, except for the casein where a micro-cracks are evident [32]. The blend between gelatin and water–glycerol molecule acts as an intrinsic protonic conductor with an ionic conductivity value σ ranging between 0.4 and 0.7 mS/cm [3,33]. The addition of the NaCl salt to the pristine gelatin blend increases the σ value. From the impedance spectra, the bulk ionic conductivity can be estimated by $\sigma =L/\left(AZ_{real}\right)$, where $Z_{real}$ is the real part of impedance when the phase angle goes to zero, L is the thickness of the gel layer and A is the area of the device [3]. Figure S1b reports the impedance spectra measured for the hydrogels as a function of NaCl content. Here, the measurement has been performed on a thin layer of hydrogel with a thickness of L = 0.2 cm and area of 5 cm^2^ contacted with two copper foils.

The cell structure is shown in the inset of Figure 1d. As can be noted, the addition of the NaCl salt leads to an enhancement of the bulk conductivity reaching a value of about 50 mS/cm at 2 M. This value is in good agreement with what has been found in the literature for gel polymer electrolytes [33,34]. Since the gelatin-based electrolyte exhibits a temperature dependence of the σ value, all the measurements have been performed at 300 K [3,35].

Choudhury et al. reported that, by further increasing the amount of salt within the hydrogel (e.g., 3 M of NaCl), a slight increment in the σ value has been observed [33]. However, a large amount of hydrated anion and cation (Cl^−^-H_2_O and Na^+^-H_2_O) negatively affects the capacitance retention and the cycle stability of the supercapacitor during the charging and discharging test [16]. A schematic representation of the accumulation and diffusion processes of the hydrated ions within the hydrogel during the operating conditions is depicted in Figure 1e. Therefore, in the present study to guarantee stable SC performance (e.g., high dielectric properties and long cycle life), a hydrogel based on gelatin with 2 M NaCl has been taken into account. It is worth noting that above this concentration, the mechanical properties of the gel deteriorate significantly and the gelation process of the GPE occurs with difficulty.

**Figure 2:**
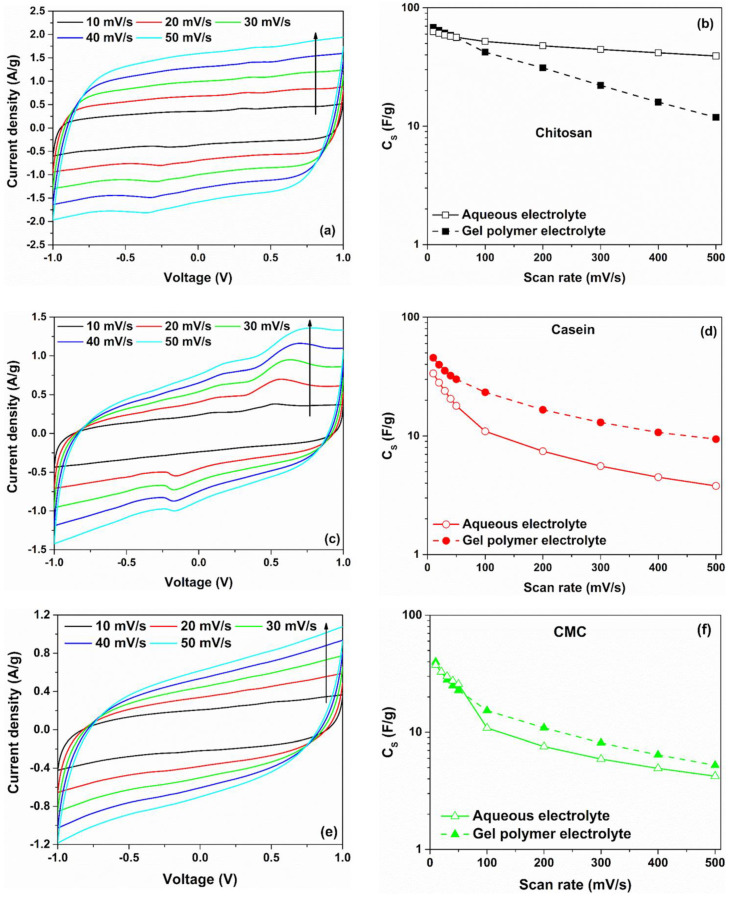
(**a**,**c**,**e**) cyclic voltammetry curves of symmetric carbon-based supercapacitors investigated in gel polymer electrolyte 2 M NaCl; (**b**,**d**,**f**) comparison of the corresponding gravimetric capacitance values between gel polymer and aqueous-based electrolytes for chitosan, casein and CMC as electrode binder, respectively.

The cyclovoltammetry curves of symmetric carbon-based supercapacitors fabricated with different binders (such as chitosan, casein and CMC) and by using a hydrogel as electrolyte are shown in Figure 2a,c,e. To avoid any chemical reactions due to the water decomposition within the GPE, the operation voltage of the SCs has been limited to a range of ±1 V [36]. The experimental data related to the CV curves for different binders, measured in the higher scan rate region (*υ* ≥ 100 mV/s), are shown in Figure S2a–c. As can be observed from Figure 2a,c,e, the investigated devices exhibit a fairly rectangular shape of the voltammetric curves at a lower scan rate region (*υ* ≤ 50 mV/s).

The clear absence of redox peaks indicates the formation of a double-layer capacitance at the interface between the electrode and hydrogel [37]. Here, the slightly slanted trend in the CV curves suggests the presence of a non-negligible ohmic contribution caused by finite conduction through the electrolyte. Moreover, the deviation from the rectangular shape for the structure having the CMC as a binder reveals a greater resistive contribution from the carbon electrode. This could lead to an increase in the equivalent series resistance for this sample [38].

By taking into account Equation (1), $C_{S}$ can be calculated from the CV curves. Figure 2b,d,f display a comparison of the $C_{S}$ values as a function of the voltage scan rates, which ranged between 10 mV/s and 500 mV/s, between the gel polymer and the reference aqueous electrolytes with NaCl as salt. For both electrolytes, the computed capacitance values decrease with the increase of *υ*. At a low scan rate (*υ* ≤ 50 mV/s), the ions have sufficient time to diffuse into the pores of activated carbon at the interface electrode/electrolyte, leading to their accumulation. This phenomenon leads to a formation of a double-layer charged at the electrodes characterized by a capacitance. As can be observed in Figure 2b, the highest value of the $C_{S}$, which is 68.2 F/g at 10 mV/s, is obtained for SC based on hydrogel with chitosan as a binder. By increasing the scan rate, the $C_{S}$ value decreases down to 11.9 F/g at 500 mV/s, corresponding to a reduction of 82.6%. 

It is worth noting that devices fabricated with commercial activated carbon materials have a specific capacitance of about 100–150 F/g (depending on the electrolyte used) [39]. Lupo et al. report values of *C_S_* ranged between 32 and 52 F/g for similar devices fabricated with chitosan as a binder and by using the same reference aqueous electrolyte [40]. By considering the casein and the CMC as binders, the reported values in the literature of $C_{S}$ ranged between 20 and 25 F/g with a percentage weight fraction (wt.%) for the AC of 90% [15,41]. These values are lower than those displayed in Figure 2d,f in the lower scan rate region. The difference can be related to the dissimilar values of the composition fraction and mass loading of the active material within the electrode. In the present study, the value of the wt.% of AC is 95%, whereas the mass loading values ranged between 6.0 and 6.8 mg/cm^2^. Moreover, supercapacitors based on casein and CMC (with hydrogel) show a value of capacitance, as a function of the scan rate, higher than those observed for the same device based on aqueous electrolyte. In particular, the electrode with the casein reports a value of 45.4 F/g, whereas the use of CMC as a binder gives a lower value of 39.8 F/g at 10 mV/s. Again, as the scan rate increases, a reduction in the dielectric properties has been observed. To quantify the drop of the capacitance as a function of the voltage scan rate, observed for all the devices investigated, the quantity $L=1-(C_{500}/C_{10})$ has been computed. Here, $C_{10}$ and $C_{500}$ correspond to the $C_{S}$ values at 10 mV/s and 500 mV/s, respectively. Figure S3 shows the percentage loss L of the capacitance $C_{S}$ as a function of the binder types for both electrolytes.

As can be seen, the SCs based on hydrogel show a more significant reduction of $C_{S}$ value of about 80% with respect to the reference system based on liquid electrolyte. This finding can be related to the lower mobility of the ions within the hydrogel resulting in a slow charge transfer and, therefore, minor ion adsorption at the electrolyte/electrode interface [16]. The difference observed for the dielectric properties (*C_S_* values) at lower (slow dynamics) and higher (fast dynamics) scan rate ranges suggests *υ*-dependent phenomena in the devices. In the supercapacitors, the charges are stored at the interface active material/electrolyte through faradaic (electron-transfer via redox reactions) processes or by the accumulation of ions at an electrical double-layer (non-faradaic processes) or by a combination of both [42]. In this latter case, the hybrid characteristics unravel the presence of a pseudocapacitive behaviour at the interface between the hydrogel and the porous carbon-based electrode. In order to distinguish between these mechanisms, the current response i(V) of the electrochemical devices can be modelled as the sum of the surface-controlled and diffusion-controlled components as $i\left(V\right)=i_{capacitive}+i_{diffusive}$, where i(V) is the current under fixed voltage [43].

**Figure 3:**
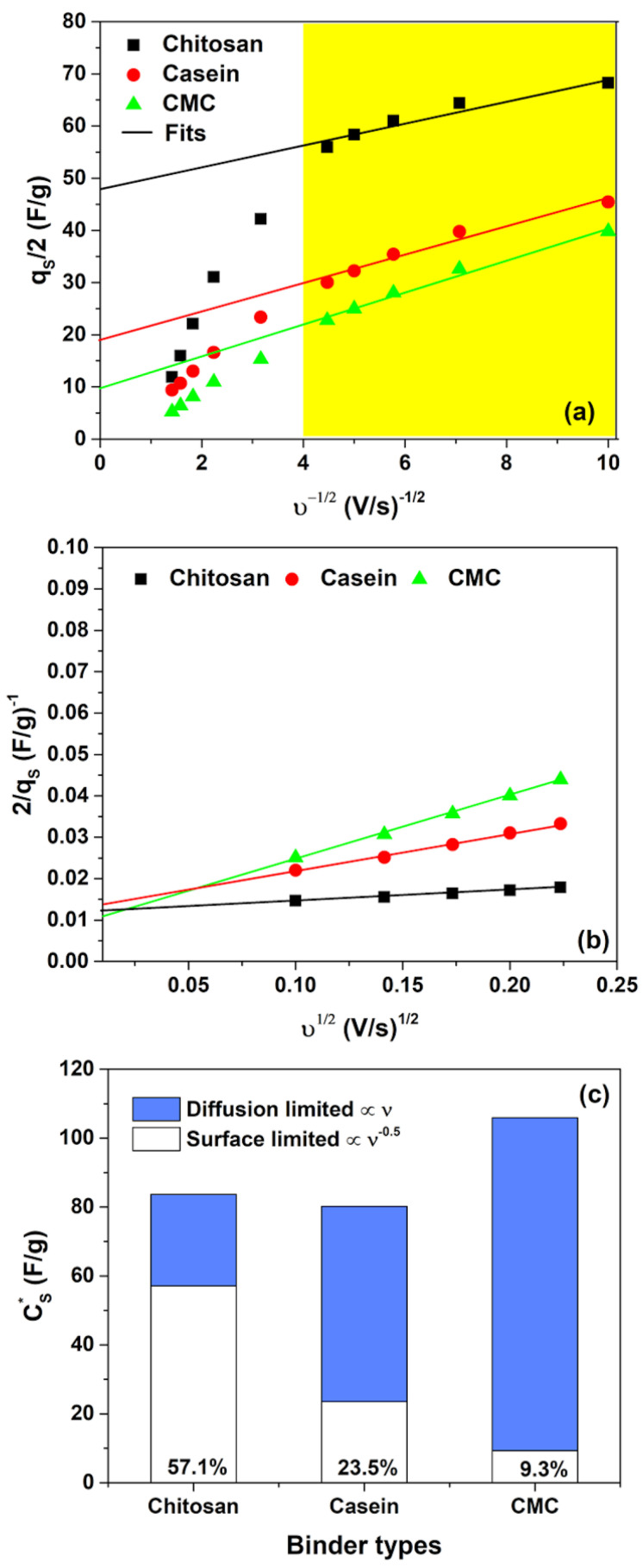
(**a**) Dependence of *q_s_* on *υ^−0.5^* and (**b**) of *1/q_s_* on *υ^0^*^.5^ for the binders investigated in hydrogel 2 M NaCl; (**c**) contribution of pseudocapacitance (diffusion-limited) and double-layer capacitance (surface-limited) to the overall capacitance $C_{S}^{*}$ for all the binders investigated. Here, *q_s_* is the total voltammetric charge. The yellow box area indicates the region where the linear fitting procedure is performed.

Here, surface limited contribution $i_{capacitive}$ is related as ∝υ, whereas the diffusion-limited contribution $i_{diffusive}$ is proportional to $\propto \upsilon^{0.5}$. Therefore, the i(V) can be written as

(5) $$i\left(V\right)=k_{1}\nu +k_{2}\upsilon^{0.5}$$

where $k_{1}$ and $k_{2}$ are constants. In order to estimate these two contributions to the overall capacitance value, Trasatti and Dunn provide a method to calculate the total charge stored by the pseudocapacitive material under study [43,44]. The total voltammetric charge $q_{S}\left(\upsilon \right)$ could be expressed as a function of scan rate through the following equation [43]

(6) $$q_{S}\left(\upsilon \right)=q_{\infty }+k\upsilon^{-0.5}$$

where $k\upsilon^{-0.5}$ represents charge storage related to semi-infinite diffusion, k is a constant and $q_{\infty }$ is the charge stored at a high scanning rate ( υ→∞). The charges stored in the double-layer, $q_{dl}$ (very similar to $q_{\infty }$), can be estimated from the intercept at *υ*-axis of $q_{s}$ vs. $\upsilon^{-0.5}$ (see Figure 3a).

Additionally, the total voltammetric charge, $q_{S}$, can be extracted from the plot of $1/q_{S}$ as a function of $\upsilon^{0.5}$ (see Figure 3b). In this framework, the pseudocapacitance charge, *q_ps_*, can be computed from the difference between $q_{S}$ and $q_{dl}$ [45]. The quantities $C_{S}^{*}$, $C_{S,dl}$ and $C_{S,ps}$ correspond to the maximum total specific capacitance at υ→0, the double-layer capacitance and pseudocapacitance, respectively. These values can be obtained by dividing the charge by the potential window of CV (i.e., 2.0 V in this work).

**Figure 4:**
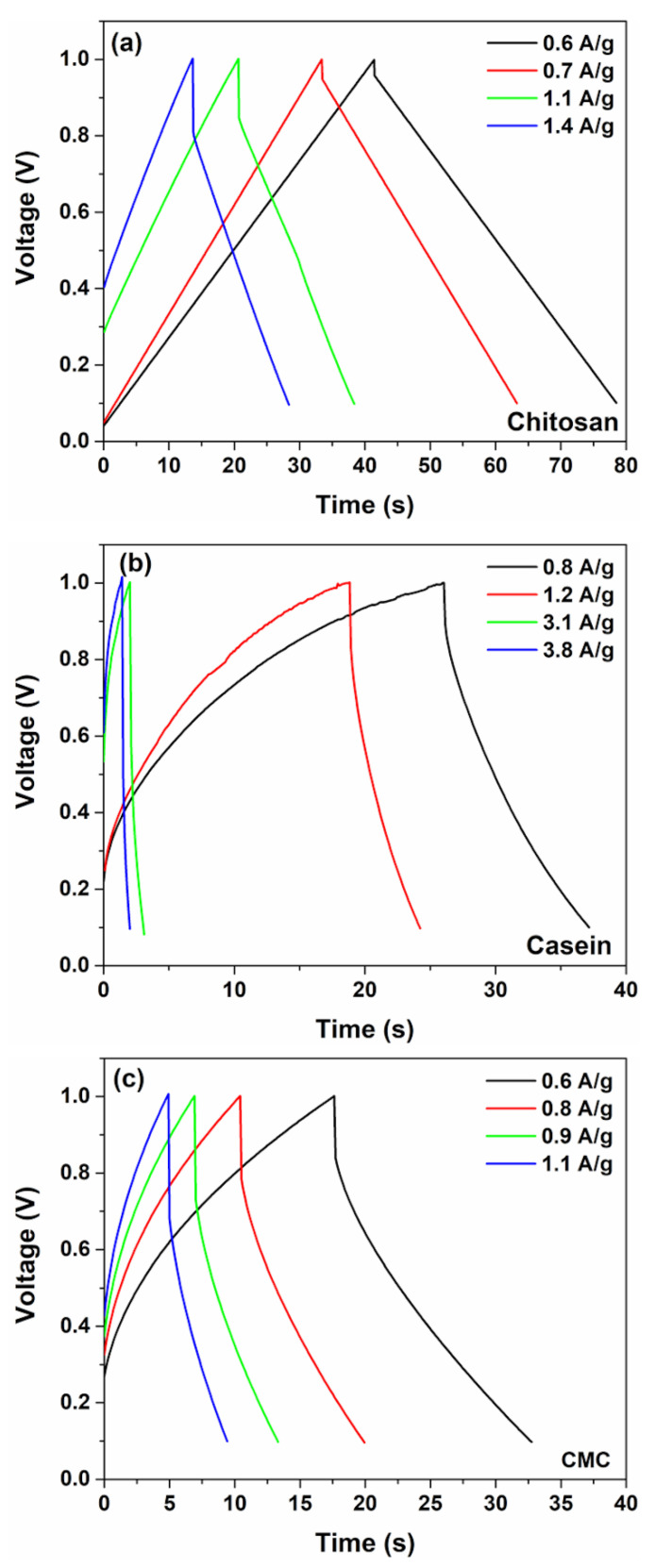
Galvanostatic charge and discharge curves at various current densities for the supercapacitors based on (**a**) chitosan, (**b**) casein and (**c**) CMC with 2 M NaCl gel polymer electrolyte, respectively.

Figure 3c shows the contributions of pseudocapacitance (diffusion-limited) and double-layer capacitance (surface-limited) to the overall capacitance $C_{S}^{*}$ for different binder types with hydrogel as electrolyte. As expected, when υ→0 the total charge is stored both with faradaic and double-layer charge storage mechanisms that occur concurrently at the electrode/hydrogel surfaces. However, only the chitosan reports a higher value of the double-layer contribution (more than 55%) to the total capacitance with respect to the other binders suggesting a substantially pure capacitance behaviour. Conversely, the electrodes based on casein and CMC show a merely pseudocapacitive behaviour, with more than 80% of $C_{S}^{*}$ originating from the fast faradaic reactions. The use of the hydrogel as an electrolyte modifies the contribution of the double-layer capacitance with respect to the SCs fabricated with the aqueous electrolyte [32], as evidenced in Figure 3c and Figure S4. It seems that the GPE promotes the pseudocapacitive behaviour assisted by charge transfer. Here, the GPE is based on a blend of gelatin and water–glycerol molecules and contains a large number of polar functional groups, which can be influenced by an electric field polarization. Moreover, gelatin is a protonic conductor and also contains a large number of divalent ions (i.e., Ca^2+^, Cu^2+^ and Fe^2+^) that can diffuse and participate in the storage mechanism. These ions can act as a dopant with electrode materials (activated carbon and binder) and give origin to pseudocapacitive behaviour. Additionally, the salt anions (Cl^−^-H_2_O) interact with the hydrophilic -OH, -COOH and -NH_2_ groups in the structure of gelatin within the hydrogel, thereby increasing solubility and the cation transport properties [34]. As expected, the observed pseudocapacitance influences also the galvanostatic charge–discharge profiles. The GCD curves measured at different current densities for the fabricated SCs are displayed in Figure 4. 

Here, for SCs with a dominant capacitive contribution of the double-layer (such as a chitosan electrode), linear charging and discharging curves have been observed. On the other hand, devices fabricated with casein and CMC report non-linear GCD curves manifested as a curvature at the beginning of the discharge profile. This behaviour can be related to the faradaic current that comes from the charge redistribution processes at the electrode surface [46].

**Figure 5:**
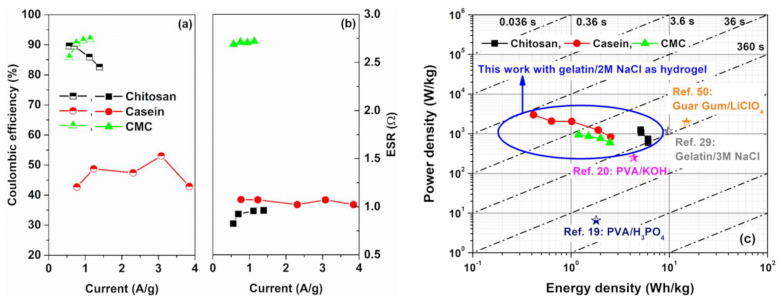
Current dependence of the (**a**) coulombic efficiency and (**b**) equivalent series resistance values as a function of the binders investigated, respectively; (**c**) Ragone plot of gravimetric power density versus gravimetric energy density for the investigated electrodes in comparison with other reported symmetric environmentally—friendly hydrogel carbon-based SCs with their associated time constant regimes [19,20,29,50].

The pseudocapacitive behaviour at the electrode/hydrogel interfaces modifies the coulombic efficiency of the devices. This quantity η can be calculated as the ratio between discharging and charging times when the charge–discharge current densities are equal. In Figure 5a, the η values computed from the GCD profiles at different current density values are shown. As can be observed, the efficiency is lower than 100% indicating that the contribution to the overall capacitance $C_{S}$ from the pseudocapacitance is not negligible [46,47]. Here, η values for the CMC and the chitosan ranged between 85 and 95%. These values are in good agreement with what is found in the literature for the same binders [32]. Conversely, the electrode based on casein shows a lower value of η of about 50%. This means that the presence of non-linear curves, related to the pseudocapacitance, is caused by an asymmetric behaviour within the device during the charging and discharging tests. Here, the capacitance value is no longer a constant during the GCD under bias current. This finding leads to a reduction in the discharge time, compared to the charging time, which negatively affects the real capability of the device to store energy efficiently [48]. 

The equivalent series resistance values of the devices can be estimated from the voltage drop observed in the GCD profiles by taking into account Equation (2). In Figure 5b, the ESR values extracted as a function of the current densities for all the supercapacitors investigated are shown. Electrodes based on chitosan and casein, which are characterized by a near-rectangular shape of the CV loop, reveal lower values of the series resistance ranging between 0.75 Ω and 1 Ω. These values are lower than one order of magnitude than those reported in the literature for similar binders [40,41]. These promising values of ESR are related to the low resistance of the electrode, due to a large amount of activated carbon within and by using a GPE with a conductivity of about 50 mS/cm.

Conversely, the device based on CMC shows an average value of the series resistance of about 2.7 Ω similar to the ESR value estimated for SC based on an aqueous electrolyte [32]. In this latter case, the greater ohmic contribution can be associated with the non-homogeneous dispersion of the conductive filler (AC) and the binder within the electrode [49]. This result confirms the behaviour of the CV loop reported in Figure 2e.

In Figure 5c, the Ragone plot, representing the specific power as a function of specific energy, for the devices under test is shown. The values of E and P have been calculated by using Equations (3) and (4), respectively. All the SCs investigated report values in good agreement with what is found in the literature for carbon-based SCs [50,51]. For comparison, in Figure S5, the Ragone plot for SCs based on the reference aqueous electrolyte is shown. As can be seen, the devices having the GPE as electrolyte show slight lower values of specific power and energy densities compared to the same SCs fabricated with an aqueous electrolyte. For both the electrolytes, the electrodes based on chitosan are the most performant devices. In particular, the SCs based on hydrogel can deliver an average value of energy and power densities of 5.2 Wh/kg and 1256 W/kg, respectively. Here, for all the binders investigated, the operating time τ=E/P ranges between 0.36 s and 36 s depending on the discharging current, as expected by the SC applications. 

In the literature, several authors report gel polymer electrolytes based on biodegradable synthetic polymer (e.g., PVA and PEO) [21,22,48] and biopolymer obtained from renewable resources (such as agar, guar gum, gelatin and starch) for supercapacitor applications [21,23,33,52,53]. However, for a reasonable comparison of the energy performance in the Ragone plot, the reference data are selected with the same potential window (0–1 V) and the same electrode material properties (e.g., activated carbon) used for the SCs fabricated. Although environmentally friendly materials have been investigated in the last decade for energy applications (e.g., Li-ion battery and supercapacitor), only a few studies are present in the literature for devices fabricated with sustainable functional materials obtained from renewable resources [40,51].

To best of our knowledge, only chitosan has been studied as a binder for fully eco-friendly supercapacitors with liquid electrolyte [32,51]. Conversely, few preliminary results have been found in the literature with the use of GPE [29]. For the other materials (e.g., CMC and casein), the literature reports devices where the electrolyte is not sustainable. Therefore, to make a comparison with the literature data for the energy performance, different devices based on GPE obtained from natural and synthetic biomaterial have been considered. 

As can be seen in Figure 5c, the chitosan-based device show an energy performance higher than those observed for the PVA based-electrolyte with H_3_PO_4_ and KOH as salts, respectively [21,22]. Choudhury et al. reported a gelatin-based electrolyte with 3 M NaCl with a value of *E* ≈ 9.7 Wh/kg slight higher than that has been reported for our devices in Figure 5b [33]. This difference can be related to the different amounts of salt that leads to an enhancement of the ionic conductivity within the blend. However, the increase in the NaCl concentration causes a faster degradation of the GPE resulting in lower cycle stability. Moreover, the use of glutaraldehyde as a crosslinking agent for collagen-based biomaterials (e.g., gelatin) increases the mechanical stability of the hydrogel but reduces its sustainability being environmentally toxic. In addition, the use of the Li salt to a GPE based on guar gum permits the fabrication of SCs with a value of specific energy density higher than 10 Wh/kg [52]. However, LiClO_4_ is very reactive and harmful to the environment and human health.

**Figure 6:**
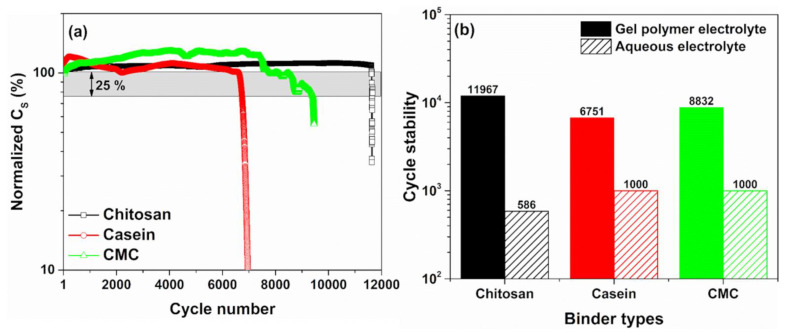
(**a**) Cycles stability of the SC-based gel polymer electrolyte 2 M of NaCl performed with CV cycles at 300 mV/s in the voltage range between 0 and 1 V; (**b**) comparison of the endurance test for the devices based on the hydrogel and the reference liquid electrolyte as a function of the binder types.

Figure 6a shows the endurance of the devices under cycle voltammetry measurements performed at 300 mV/s and in the voltage range between 0 and 1 V. It is worth noting that the capacitance values measured for the SCs investigated are in good agreement with what has been reported in Figure 2. As evidenced, the device with a dominant contribution of the double-layer capacitance shows a stable behaviour of the dielectric properties up to 12,000 working cycles (e.g., chitosan as a binder). On the other hand, the casein and the CMC-based supercapacitors, where the contribution of the faradaic reaction at the interface is most significant, exhibit lower cycle stability estimated at 8832 and 6751, respectively. These cycle number values are estimated by considering a reduction of 25% from its initial capacitance value. Moreover, all the devices with hydrogel show an enhancement of the dielectric properties ranging between 10 and 30% during the cycling test. This trend has been already observed for the casein and the CMC as binder immersed in an aqueous electrolyte [32]. This finding is a further evidence that faradaic reactions at the electrode interface are supported when the GPE is used. 

Figure 6b displays the comparison to the cycling test for the devices based on the hydrogel and the reference liquid electrolyte as a function of the binder types. As can be noted, the use of the GPE permits to increase the cycle stability of the devices exceeding the 1000 cycles reported for the reference devices based on liquid electrolyte having similar electrode properties. In Figure S6, the cycles stability properties for the SCs reported in the literature based on eco-friendly GPE are shown. For comparison, only the reference SCs based on PVA/KOH shows value of the cycle stability in the range of 10,000 working cycles. The other devices based on PVA/H_3_PO_4_ and guar gum/LiClO_4_ hydrogels have a lower endurance (≤5000 cycles). Additionally, the hydrogel based on 3 M of NaCl displays lower stability down to 1200 cycles. It should be noted that in the literature, supercapacitors based on biopolymer hydrogel electrolytes with different electrode fillers (such as graphene, MnO_2_ and carbon nanotubes), conducting salts (e.g., Na_2_SO_4_, Li_2_SO_4_ and LiCl) and aqueous electrolyte (e.g, H_2_SO_4_ and KOH) have values of stability lower than 10,000 cycles [16,34]. Although electrolytes and salts that contain sulphur atoms are expected to be benign for the environment, after combustion, they emit SO_2_ that contributes to acid rain. Therefore, they cannot be considered entirely eco-friendly [54].

To investigate the correlation between the dielectric properties and the charge storage mechanisms within the supercapacitors for both the electrolytes, electrochemical impedance spectra measurements have been performed. This non-destructive technique has been extensively used in the literature for electrochemistry and energy applications from generation to storage energy [42,55,56,57].

**Figure 7:**
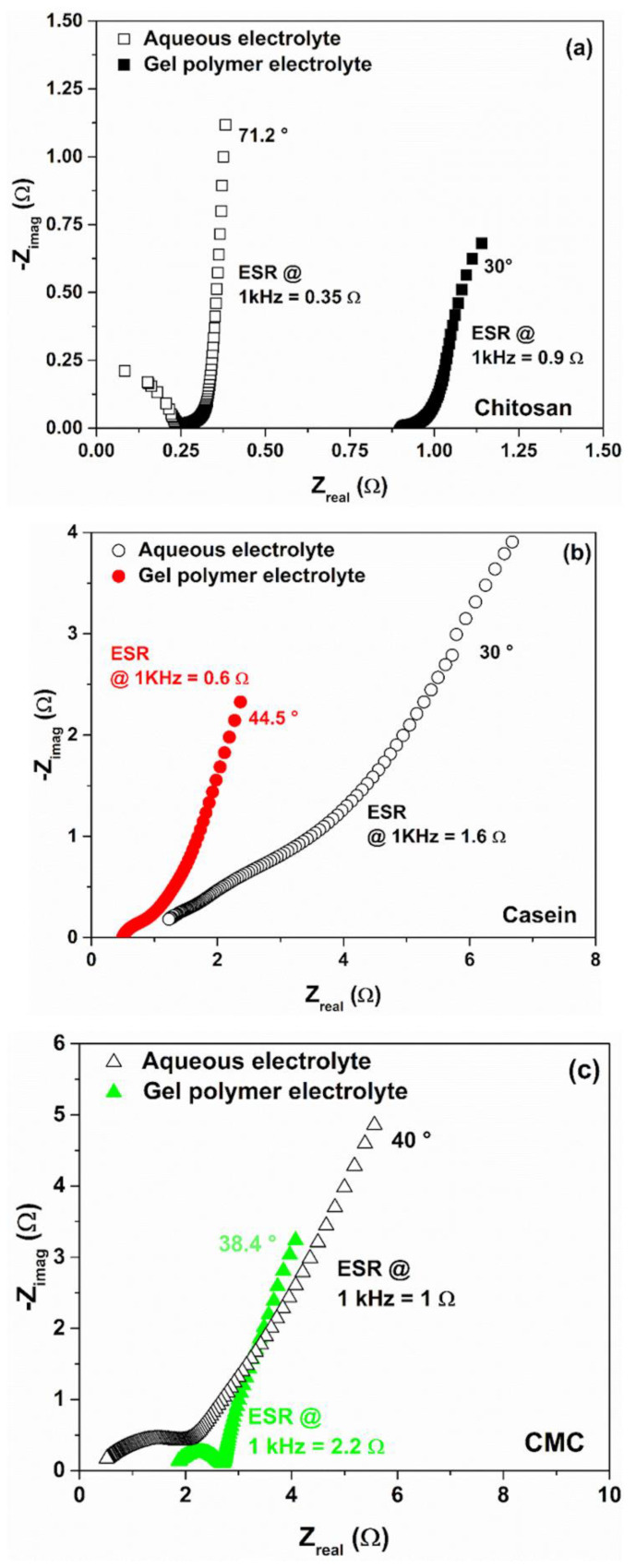
Comparative Nyquist plots for the supercapacitors based on gel polymer and reference liquid electrolytes with (**a**) chitosan, (**b**) casein and (**c**) CMC as an electrode binder.

Figure 7 reports the Nyquist plots representing the imaginary part, -*Z_imag_*, as a function of the real part, *Z_real_*, of the complex impedance for the binders and electrolytes taken into account. As expected for the supercapacitors, all the spectra have a long tail at lower frequencies which is a typical shape observed for the charge storage mechanisms of the capacitive and pseudocapacitive materials and their associated interfacial phenomena [42].

In the literature, several studies report electrochemical impedance spectroscopy models that describe the interface kinetics between the porous electrodes and the electrolytes [42,56,58,59].

Devices characterized with a pure capacitive behaviour (only charge accumulation without any transfer) show a simple vertical line that can be modelled as a series combination of resistive and capacitive elements. Here, the delay angle of the imaginary part of the impedance approaches the theoretical value of 90°. A small difference to the ideal case can be attributed to the porosity of the carbon-based electrode [42,60].

Conversely, when faradaic reactions occur at the surface, a second slope of the imaginary part of the impedance in the low-frequency region has been reported [56,58]. For all the devices fabricated, the resulting value of the frequency shift decreases in a range between 30° and 44.5°, confirming the presence of a major pseudocapacitive contribution arising from the diffusion [27]. This behaviour can be related to the presence of a large number of polar functional groups within the gelatin that can be influenced by the electric field polarization [61]. Moreover, the pseudocapacitance behaviour observed at the electrode interface is also influenced by the binders that contain atoms (such as Na in casein and CMC) and functional groups (e.g., carboxyl, hydroxyl and amino) that interact with the activated carbon at the interface with the gel electrolyte [32]. Therefore, the appearance of a semicircle in the spectra can be ascribed to these interactions [42,59]. In this framework, the diffusion-limited/capacitive response can be easily described and modelled by a Randles equivalent circuit model [28].

As evidenced by the ESR values extracted at 1 kHz from the impedance spectra in Figure 7, the use of the GPE in place of the liquid electrolyte produces an increase in the ohmic contribution for the chitosan and the CMC binders. Here, the devices show experimental spectra shifted towards a higher resistance range (1–4 Ω). This means that a diffusion layer near the electrode interface is present with a non-negligible resistance value [62].

It is worth noting that only for the casein, the SC with hydrogel shows lower ohmic contribution with respect to the same device with reference liquid electrolyte. This finding is in good agreement with what has been reported in Figure 2d and confirms the better dielectric properties observed for the GPE sample. Moreover, the device based on CMC shows a clear semicircle loop with a diameter of about 1 Ω, lower than that observed for the liquid electrolyte (1.9 Ω), suggesting an increase in the exchange current from the charge-transfer processes at the electrode/hydrogel interface [63].

**Figure 8:**
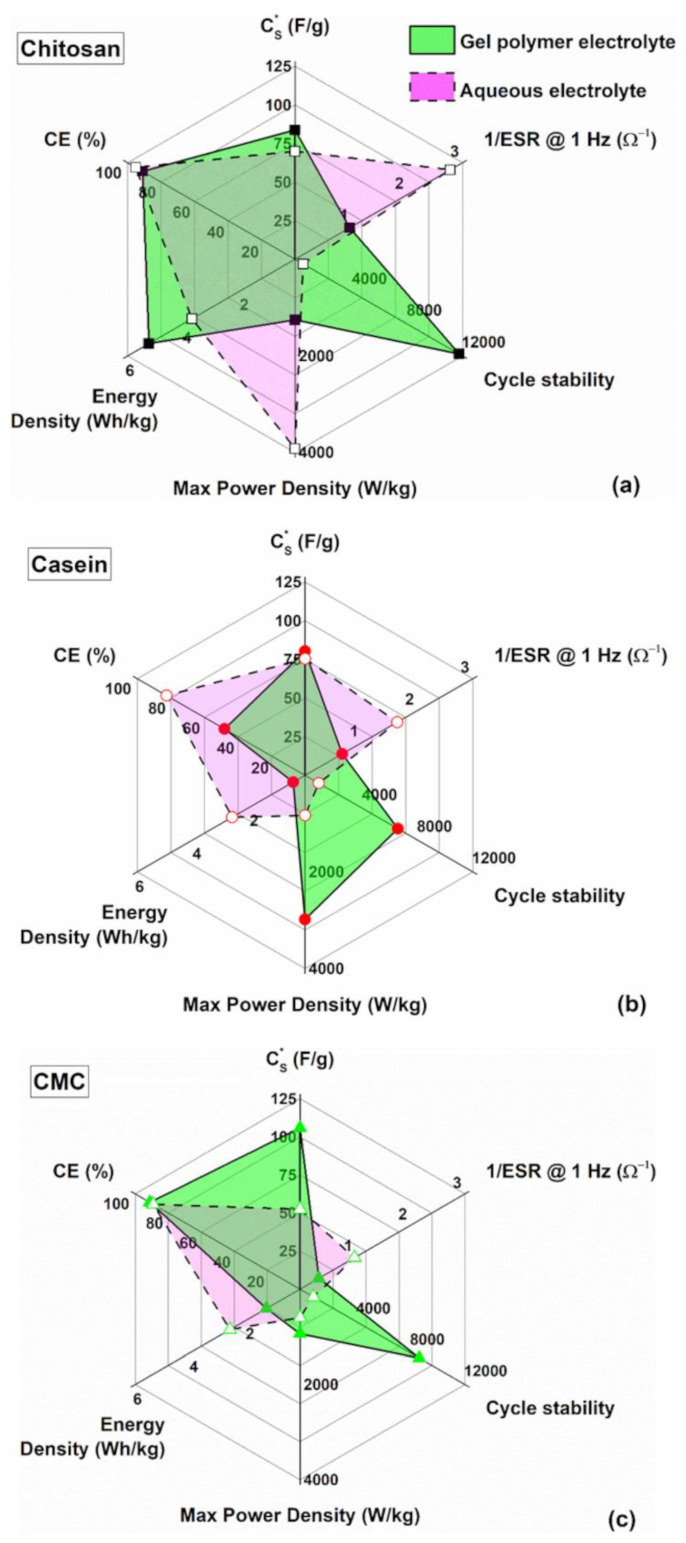
Radar plot to compare the performance of the supercapacitors based on gel polymer and reference liquid electrolytes with (**a**) chitosan, (**b**) casein and (**c**) CMC as an electrode binder.

In Figure 8a, a comparison in terms of the lifespan (cyclic number), energy and dielectric performances of the investigated supercapacitor by varying the binder types and for both the electrolytes is reported. Aqueous electrolytes are superior, compared the other ones, in terms of their ionic conductivity, interfacial wettability, safety and environmentally benign nature [16]. The experimental data indicate that hydrogel enhances the stability of the final device increasing the endurance up to 12,000 cycles (e.g., for chitosan as binder with GPE). Although the internal resistance increases with the gel-like electrolyte the dielectric properties, in terms of specific capacitance and energy performances, still remain. Here, the chitosan used as a binder shows better properties compared to the other binders with hydrogel as an electrolyte.

## 4. Conclusions

Hydrogel based on gelatin and doped with 2 M of NaCl has been used as a gel polymer electrolyte in symmetric carbon-based supercapacitors with sustainable electrodes. Biodegradable materials obtained from renewable resources such as chitosan, casein and carboxymethyl cellulose have been employed as binders within the electrode in combination with activated carbon extracted from the coconut shell. In order to evaluate the influence of the hydrogel on the device performance, reference supercapacitors have been fabricated with the aqueous electrolyte with 1 M of NaCl for all the binders investigated.

The most performant supercapacitors with gel-like electrolytes are characterized by a gravimetric capacitance value ranging between 80 and 100 F/g, a series resistance contribution lower than 1 Ω and good coulombic efficiency. However, a marked capacitance loss as a function of the voltage scan rate has been observed for all the binders taken into account. By using the partition method, pseudocapacitance behaviour has been found at the electrode/electrolyte interface. As evidenced, different charge storage mechanisms take place within the SCs where the surface and diffusion-limited processes are concurrent at the electrode interface.

Compared to the devices with reference aqueous electrolytes, supercapacitors based on hydrogel show a major contribution of the diffusion component to the dielectric response. The diffusion component also influences the impedance spectra where a long tail at a low-frequency region in the Nyquist plots has been observed. Here, chitosan-based devices show a capacitive-like dielectric response similar to an electrochemical double-layer capacitor. Conversely, for the CMC and casein-carbon-based electrodes, the diffusion contribution to the overall capacitance is dominant. 

In terms of cyclability, the hydrogel enhances the cycle life of the supercapacitor showing an endurance higher than 12,000 cycles (without attenuation) for the chitosan-based electrode. A similar trend has been found for both the casein and CMC binders showing cycle stabilities up to 7000 and 9000, respectively. The best-performing device can deliver ca. 5.2 Wh/kg of energy at a high power density of 1256 W/kg.

## Data Availability

The data presented in this study are available on request from the corresponding author.

## Supplementary Materials

The following supporting information can be downloaded at https://www.mdpi.com/article/10.3390/polym14204445/s1, Figure S1: (a) Cross-section of the symmetric carbon-based supercapacitors with the corresponding chemical structures of the sustainable materials used as binder and polymer electrolyte. (b) Nyquist plots for the pristine and the doped hydrogels as a function of the NaCl content; Figure S2: Cyclic voltammetry curves of symmetric carbon-based supercapacitors investigated in gel polymer electrolyte 2 M NaCl for (a) chitosan, (b) casein and (c) CMC as electrode binder. The voltage scan rate is ranged between 100 and 500 mV/s; Figure S3: Capacitance percentage loss as a function of the binder types for aqueous and gel polymer electrolytes; Figure S4: Contribution of pseudocapacitance (diffusion-limited) and double layer capacitance (surface-limited) to the overall capacitance $C_{S}^{*}$ for all the binders investigated with aqueous electrolyte; Figure S5: Ragone plot of gravimetric power density versus gravimetric energy density for the investigated electrode with aqueous electrolyte; Figure S6: Cycles stability for the SCs reported in the literature based on gel-polymer electrolyte.
