# Supplementary material for: Electrochemical Performance of Biopolymer-Based Hydrogel Electrolyte for Supercapacitors with Eco-Friendly Binders
Source: Polymers (Basel). 2022 Oct 20;14(20):4445. doi: 10.3390/polym14204445 (PMC9608640; doi:10.3390/polym14204445)
Supplement: Supplementary file 1 [file polymers-14-04445-s001.zip › polymers-1964358-supplementary.pdf]

polymers

Supporting information

Electrochemical performance of biopolymer-based hydrogel electrolyte for supercapacitors with eco-friendly binders

Giovanni Landi *, Luca La Notte , Alessandro Lorenzo Palma and Giovanni Puglisi

ENEA, Casaccia Research Center, Via Anguillarese 301, 00123 Rome, Italy
* Correspondence: giovanni.landi@enea.it; Tel.: 0039-0817723627

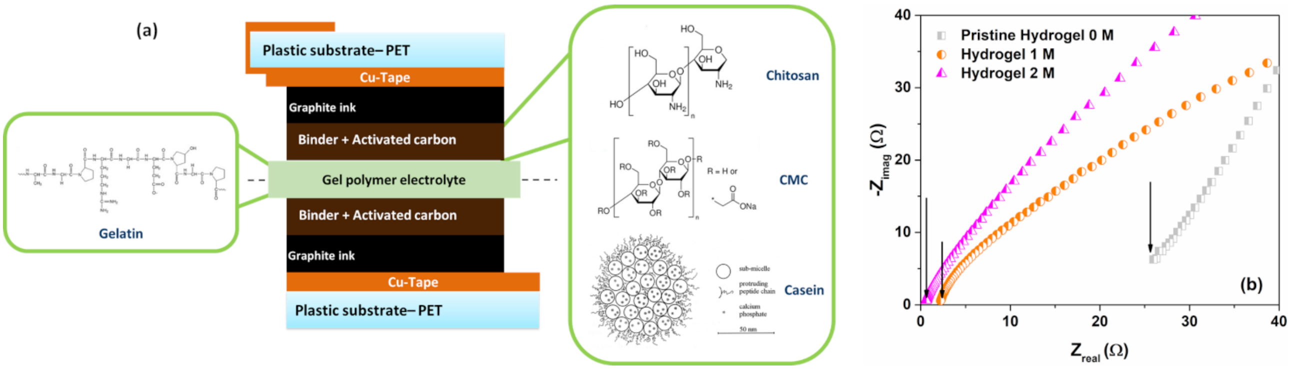

Figure S1. (a) Cross-section of the symmetric carbon-based supercapacitors with the corresponding chemical structures of the sustainable materials used as binder and polymer electrolyte. (b) Nyquist plots for the pristine and the doped hydrogels as a function of the NaCl content.

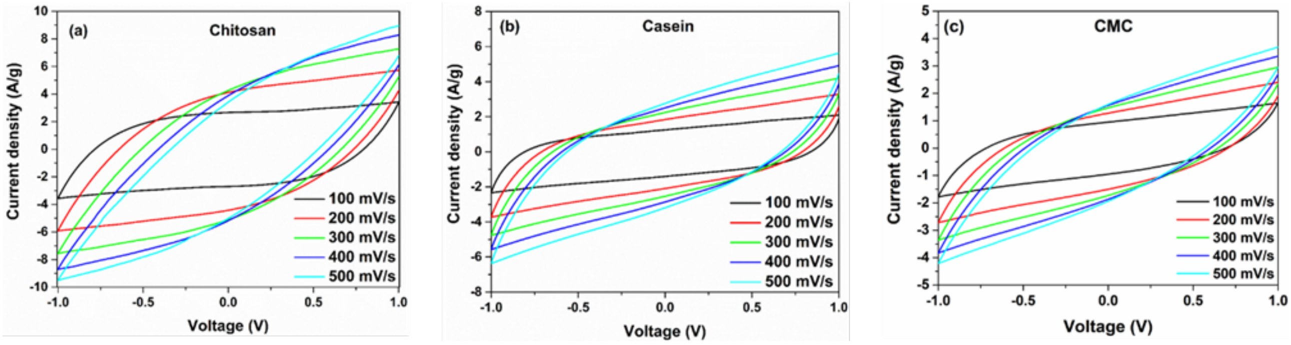

Figure S2. Cyclic voltammetry curves of symmetric carbon-based supercapacitors investigated in gel polymer electrolyte 2 M NaCl for (a) chitosan, (b) casein and (c) CMC as electrode binder, respectively. The voltage scan rate is ranged between 100 and 500 mV/s.

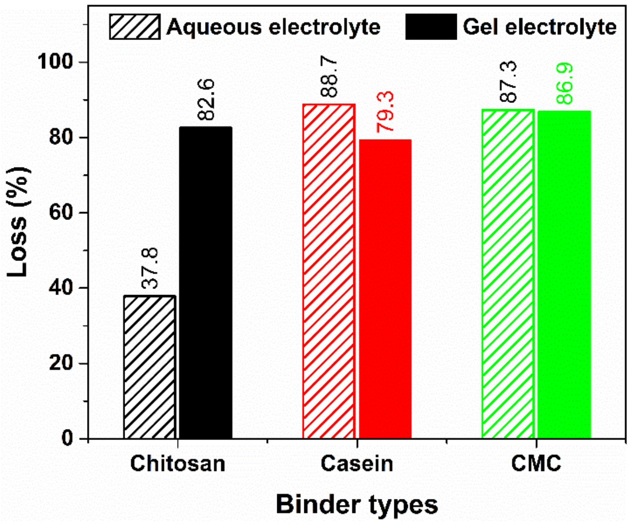

Figure S3. Capacitance percentage loss as a function of the binder types for aqueous and gel polymer electrolytes, respectively.

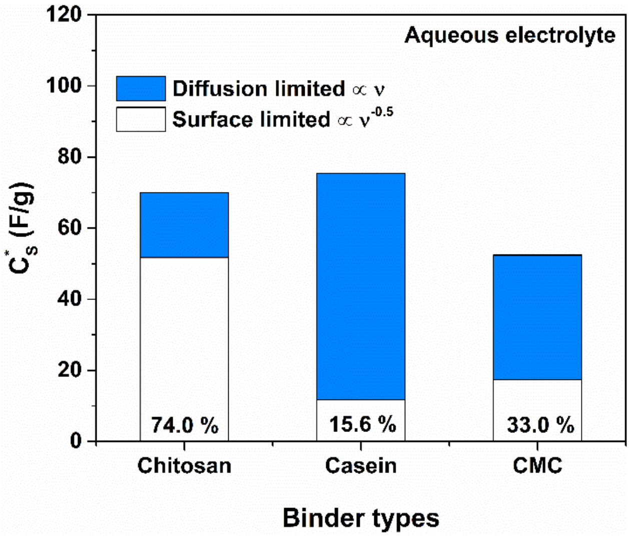

Figure S4. Contribution of pseudocapacitance (diffusion-limited) and double layer capacitance (surface-limited) to the overall capacitance C_S^* for all the binders investigated with aqueous electrolyte.

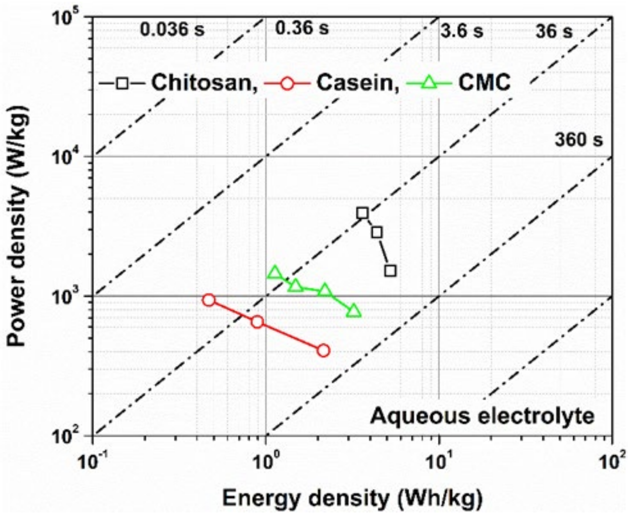

Figure S5. Ragone plot of gravimetric power density versus gravimetric energy density for the investigated electrode with aqueous electrolyte.

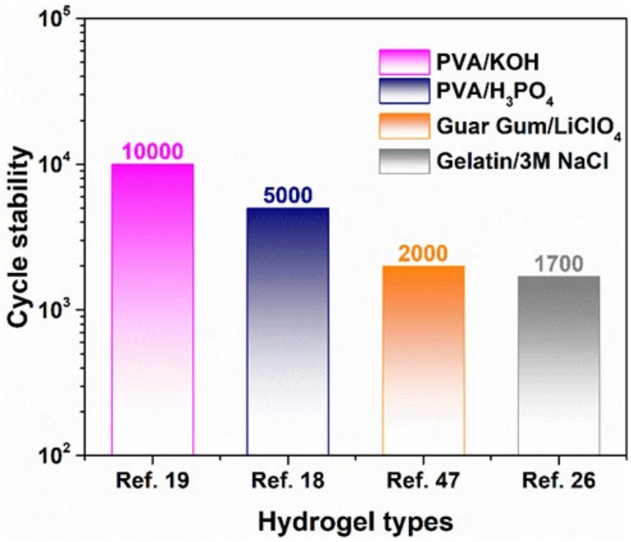

Figure S6. Cycles stability for the SCs reported in the literature based on gel-polymer electrolyte.
